# Correction to “Synthesis and Characterization of Dy_2_O_3_@TiO_2_ Nanocomposites for Enhanced Photocatalytic and Electrocatalytic Applications”

**DOI:** 10.1021/acsengineeringau.5c00015

**Published:** 2025-05-06

**Authors:** Balachandran Subramanian, K. Jeeva Jothi, Mohamedazeem M. Mohideen, R. Karthikeyan, A. Santhana Krishna Kumar, Ganeshraja Ayyakannu Sundaram, K. Thirumalai, Munirah D. Albaqami, Saikh Mohammad, M. Swaminathan

There is an unintentional error in Figure 3 in the original article in which a TEM image is inappropriately copy-pasted from the TEM raw image folder. This correction addresses the image duplication within Figure 3. For this change, there are no changes in scientific content or interpretations or any results.

**Figure 3:**
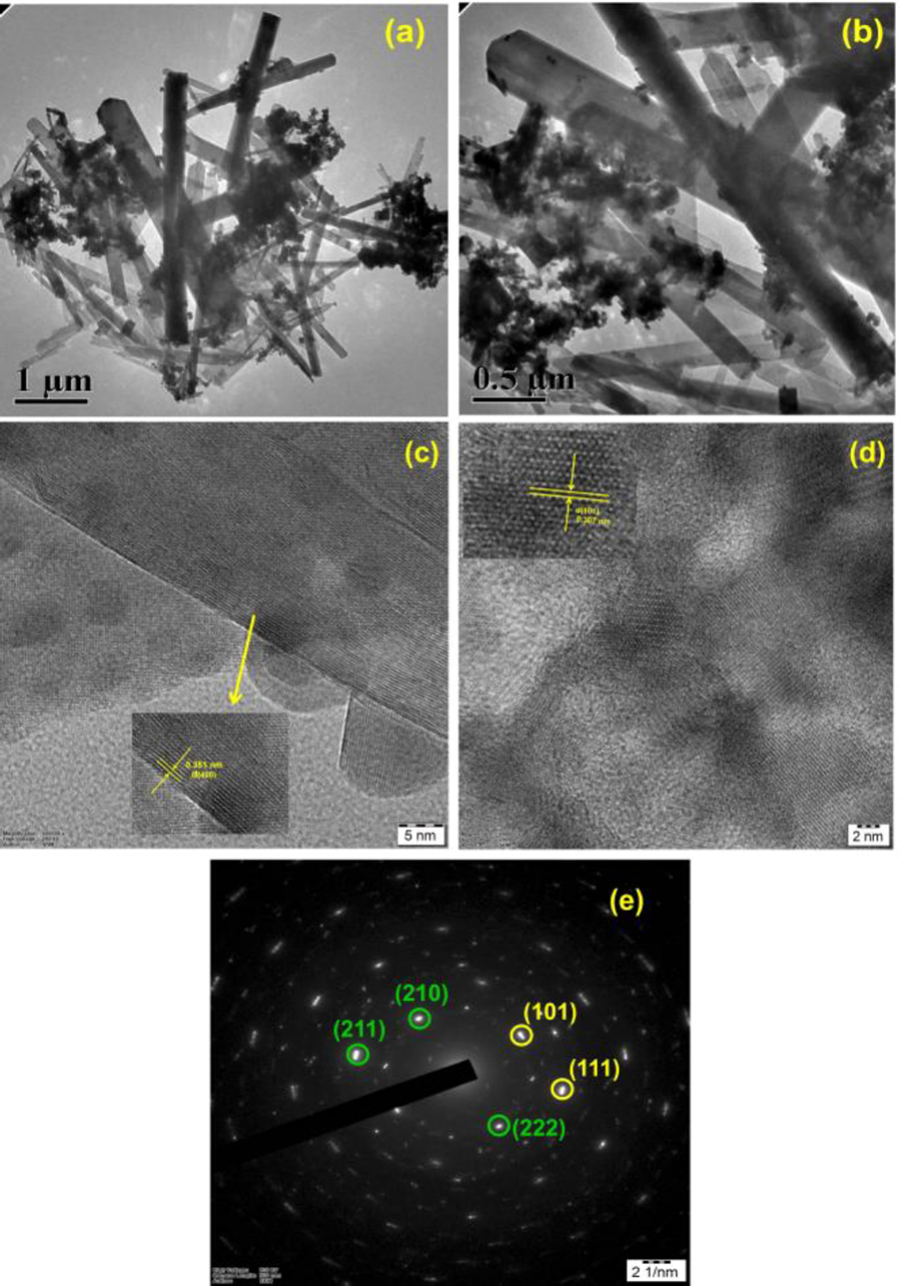
Low-magnification TEM images of 3 wt % of Dy_2_O_3_@TiO_2_ micro rods and intercrossed sheet distributions: (a) 1 μm and (b) 0.5 μm; (c and d) high-magnification TEM image of Dy_2_O_3_-modified TiO_2_ lattice fringes (5 and 2 nm); (e) corresponding SAED pattern (2 1/nm).

